# A Rare Case of Weil’s Disease Presenting With Severe Hyperbilirubinemia and Acute Kidney Injury: A Diagnostic Challenge in Urban England

**DOI:** 10.7759/cureus.73700

**Published:** 2024-11-14

**Authors:** Mohammed Ibrahim Salih Elfaki, Raumesha Biladiwala

**Affiliations:** 1 Internal Medicine, Health Education England - North East, Newcastle-Upon-Tyne, GBR; 2 Internal Medicine, Northumbria Healthcare NHS Trust, Newcastle-Upon-Tyne, GBR

**Keywords:** acute fulminant hepatitis, febrile jaundice, hyperbilirubinaemia, severe leptospirosis, weil's disease

## Abstract

We present a rare and challenging case of Weil’s disease in a patient who was admitted to the Critical Care Unit with a clinical suspicion of the condition. The patient showed a prompt response to treatment before laboratory confirmation was obtained. Leptospirosis, a zoonotic disease, is most commonly found in tropical and temperate regions. While it is uncommon in the UK, it carries the potential for severe complications if not treated promptly. We report a 57-year-old gentleman from the North of England with no previous past medical history of note who presented with febrile illness and jaundice. His symptoms included fever, abdominal pain, and poor eating and drinking. Investigations showed raised inflammatory markers and deranged liver function tests (LFTs) with a hepatitic picture. Common causes of deranged LFTs including viral hepatitis, excessive alcohol intake, and paracetamol poisoning were excluded. In addition, CT abdomen and magnetic resonance cholangiopancreatography (MRCP) showed no obstructive lesions. He was admitted to the Intensive Treatment Unit (ITU) for monitoring due to impending multiorgan dysfunction. More elaborative history suggested leptospirosis, and he was treated empirically with intravenous ceftriaxone. Leptospira IgM and polymerase chain reaction (PCR) came back positive indicative of severe leptospirosis. He improved remarkably while in the ITU and was discharged to the ward and then home a few days later. In conclusion, we want to highlight leptospirosis as a cause of fulminant acute hepatitis with potential multiorgan failure and should be suspected even in urban regions when other common causes of hepatitis are excluded.

## Introduction

Leptospirosis is a zoonotic disease caused by the spirochete *Leptospira*. Other well-known spirochetal diseases include syphilis and Lyme disease. The incubation period for leptospirosis typically ranges from 7 to 13 days but can extend from 2 to 20 days in some cases [1]. Leptospirosis remains a significant global public health concern, particularly in tropical and subtropical regions. The disease is endemic in both urban and rural environments, with transmission primarily occurring through contact with water or soil contaminated by *Leptospira *organisms [2]. Recent studies have reported an increasing incidence and geographic expansion of leptospirosis, underscoring the need for enhanced surveillance and preventive measures [1]. Clinically, leptospirosis presents with a wide spectrum of symptoms, from asymptomatic infections to severe forms such as Weil’s disease and severe pulmonary haemorrhage syndrome (SPHS). Initial symptoms often include fever, headache, myalgia, and conjunctival suffusion, which can lead to misdiagnosis due to overlap with other febrile illnesses. In severe cases, multi-organ involvement may occur, including hepatic and renal dysfunction, pulmonary haemorrhage, and cardiovascular collapse [3]. Early recognition of leptospirosis is essential for the timely initiation of antimicrobial therapy, which has been shown to reduce the disease's severity and duration. However, diagnosing leptospirosis remains challenging, especially in resource-limited settings where access to serological and molecular diagnostic tests is limited. Novel diagnostic tools, including rapid point-of-care tests, are currently under development and promise to improve early detection and management [4]. Leptospirosis can lead to various complications, significantly affecting patient outcomes. Renal involvement, typically presenting as acute kidney injury and tubulointerstitial nephritis, is one of the most common complications and may result in long-term sequelae, including chronic kidney disease. Pulmonary complications, such as acute respiratory distress syndrome (ARDS) and SPHS, pose significant management challenges and are associated with high mortality rates [3]. In this study, we present a rare case of Weil’s disease accompanied by severe hyperbilirubinemia. A literature review on this subject is also provided to raise awareness of the gastrointestinal manifestations of the disease and its potential complications.

## Case presentation

A 57-year-old gentleman, previously well, with no significant medical history, and "had not seen a doctor in years", presented to the hospital with a 10-day history of feeling generally unwell, accompanied by intermittent fever. He also reported poor appetite, dark urine, and unintentional weight loss of approximately 7 kg over the past two months. The patient denied any increase in alcohol consumption, overdose (including paracetamol), or recreational drug use. On examination, the patient was profoundly jaundiced but showed no signs of hepatic encephalopathy or organomegaly, and his physical examination was otherwise unremarkable, apart from minor excoriation marks on his lower legs. Blood samples were obtained and sent for analysis. A venous blood gas test returned normal results. Given the strong clinical suspicion of obstructive jaundice, a Computerized Tomography (CT) scan of the abdomen was performed in the Emergency Department. The scan revealed a mildly enlarged head of the pancreas, but there was no ductal dilation or identifiable lesion to explain the findings. 

In the meantime, the laboratory results revealed severe acute kidney injury (AKI stage 3), with a creatinine level of 583 µmol/L (baseline 94 µmol/L from three years prior) and urea of 71.6 mmol/L. Liver function tests showed significant derangement, with a markedly elevated bilirubin level of 814 µmol/L (47.6 mg/dL), alanine aminotransferase (ALT) of 384 U/L, and alkaline phosphatase (ALP) of 238 U/L. Surprisingly, despite the potential for multi-organ failure, the patient appeared relatively well clinically. Table 1 summarizes the initial blood test results.

**Table 1:** Key blood test results indicating severe acute kidney injury and liver dysfunction.

Test	Result	Reference Range
Creatinine	583 µmol/L	60-110 µmol/L
Urea	71.6 mmol/L	2.5-7.8 mmol/L
Bilirubin	814 µmol/L (47.6 mg/dL)	3-17 µmol/L
Amino Aminotranferase (ALT)	384 U/L	7-56 U/L
Alkaline Phophatase (ALP)	238 U/L	44-147 U/L

Given the significantly deranged blood tests and risk of impending multi-organ failure, the patient was admitted to the Critical Care Unit for closer monitoring and potential organ support. In the interim, further investigations, including viral serology, paracetamol levels, and an autoimmune liver, returned negative results. The case was discussed with the gastroenterology team, who recommended performing a magnetic resonance cholangiopancreatography (MRCP) to obtain a clearer view of the hepatobiliary system, despite the lack of clear evidence suggesting obstruction. As expected, the MRCP was unremarkable, showing no obstruction or suspicious lesions.

Further history obtained during this admission revealed that the patient maintained a small garden, which he reported was heavily infested with rats. This detail, along with the exclusion of other common causes of acute liver and kidney dysfunction, raised clinical suspicion for leptospirosis. Given the diagnostic challenge and history raising suspicion for leptospirosis, a Gastroenterology opinion suggested initiating empirical intravenous ceftriaxone, and subsequently, Leptospira IgM and polymerase chain reaction (PCR) tests were requested.

While in the Critical Care Unit, the patient remained clinically stable, with no need for vasopressors or renal replacement therapy. Although his kidney function indicated severe acute kidney injury, dialysis was not required as the patient continued to maintain a reasonable urine output. Remarkably, within the first 48 hours of starting ceftriaxone, the patient demonstrated significant improvement in both renal and liver function. His oral intake improved, and clinically, the jaundice began to resolve. Table 2 shows blood tests while in hospital. Later on admission, Leptospirosis IgM and PCR results returned positive, confirming the diagnosis of Weil’s disease. The patient continued to improve and was discharged home a few days later on oral doxycycline.

**Table 2:** Blood tests showing significant improvement to ceftriaxone treatment

Test	Reference range	Day 0 (admission)	Day 1	Day 2	Day 3	Day 5	Day 7
Bilirubin	3-17 µmol/L	814	634	488	231	154	149
Creatinine	60-110 µmol/L	583	391	281	167	129	130
Urea	2.5-7.8 mmol/L	71.6	54.8	38.5	22.9	17.4	14.6

## Discussion

Leptospirosis is a zoonotic disease historically known to affect tropical and subtropical regions. In Europe, the incidence of leptospirosis decreased during the second half of the last century leading to a decrease in disease awareness and recognition. However, reports suggest that leptospirosis is re-emerging particularly in urban industrialised areas. Although the incidence of leptospirosis is rising in Europe, it has been regarded as a neglected disease [5]. It is to be noted that the reports indicate the disease pattern in Europe is different from the classical natural history of Leptospirosis. This fact, besides the relatively non-specific symptoms and the lack of availability of laboratory tests, might make diagnosing leptospirosis challenging. Early symptoms such as fever, myalgia, and headache are non-specific and overlap with illnesses like influenza, viral hepatitis, or sepsis. In severe cases, complications like Weil’s disease or severe pulmonary hemorrhagic syndrome (SPHS) may mimic other critical conditions, further delaying diagnosis. Leptospirosis is well-known to occur in tropical regions and mostly in rural regions. In Europe, the disease is noted to occur in urban regions, a phenomenon not well understood [2]. The mode of transmission is through direct contact with infected rat urine and can occur indirectly through contaminated soil or water. Infection by rat urine is the more frequent form of infection [6].

Our patient is a middle-aged man with no history of significance. He lives in an urban area, not from a historically high-risk location or job. This correlates well with another leptospirosis case report in a patient who works in a prison kitchen with poor sanitary conditions [7]. As such, a relatively rare diagnosis of leptospirosis was not initially suspected. An important clue to the diagnosis is the fact that he has a small garden which he reported to be heavily infested by rats. This was only sought after full sets of investigations for common causes of deranged liver function tests were excluded. It was noted that the patient looked well clinically despite severely deranged blood tests. A striking feature we noticed is the extremely high level of hyperblilirubinaemia. We have noted previous rare reports of such severe hyperbilirubinemia [8]. Our patient was transferred to the intensive treatment unit (ITU) due to impending multiorgan dysfunction and was started on empirical treatment for leptospirosis while awaiting laboratory confirmation. In the subsequent days, a significant response to treatment was noted with a fall of bilirubin by approximately 200 µmol/L/day and an improvement in renal function. The patient did not need organ support and was deemed fit for the ward and discharged home a few days later.

An essential educational takeaway is the importance of maintaining a broad differential diagnosis and considering alternative possibilities. Additionally, this case underscores the critical role of thorough patient history-taking; even minor details can significantly influence patient outcomes, and relying solely on investigations may not suffice to ensure accurate diagnosis and treatment.

## Conclusions

In conclusion, this case report highlights both the typical and atypical aspects of leptospirosis presentation, emphasizing the diagnostic challenges posed in urban, non-endemic settings. While our patient exhibited hallmark signs of severe leptospirosis, including jaundice, acute kidney injury, and hyperbilirubinemia consistent with Weil’s disease, several features diverged from the classical presentation. Notably, the patient’s relatively mild systemic symptoms, despite profound biochemical abnormalities, and his urban residential setting without overt occupational risk factors, contributed to the initial diagnostic uncertainty. This underscores the need for heightened clinical suspicion even in atypical contexts, such as urban environments, where leptospirosis may not be immediately considered.

Furthermore, the unusually high level of hyperbilirubinemia observed in this patient aligns with rare but documented severe presentations of the disease, offering additional insight into its clinical spectrum. The case demonstrates the importance of integrating thorough environmental history-taking, including seemingly minor details like rodent exposure in domestic gardens, which ultimately led to the correct diagnosis. By illustrating both the common and uncommon elements of leptospirosis, this report underscores the need for clinicians to maintain a broad differential diagnosis and consider leptospirosis even in urban and industrialized settings. Enhanced awareness, combined with improved diagnostic tools, will be crucial in managing this re-emerging zoonotic disease, ensuring timely recognition and treatment to prevent severe outcomes.
